# Interfocal heterogeneity challenges the clinical usefulness of molecular classification of primary prostate cancer

**DOI:** 10.1038/s41598-019-49964-7

**Published:** 2019-09-19

**Authors:** Kristina Totland Carm, Andreas M. Hoff, Anne Cathrine Bakken, Ulrika Axcrona, Karol Axcrona, Ragnhild A. Lothe, Rolf I. Skotheim, Marthe Løvf

**Affiliations:** 1 Department of Molecular Oncology, Institute for Cancer Research, Oslo University Hospital-Radiumhospitalet, Oslo, Norway; 2 Institute for Clinical Medicine, Faculty of Medicine, University of Oslo, Oslo, Norway; 3 Department of Pathology, Oslo University Hospital-Radiumhospitalet, Oslo, Norway; 4 Department of Urology, Akershus University Hospital, Lørenskog, Norway; 5 Department of Informatics, Faculty of Mathematics and Natural Sciences, University of Oslo, Oslo, Norway

**Keywords:** Cancer genomics, Tumour heterogeneity, Prostate cancer

## Abstract

Prostate cancer is a highly heterogeneous disease and typically multiple distinct cancer foci are present at primary diagnosis. Molecular classification of prostate cancer can potentially aid the precision of diagnosis and treatment. A promising genomic classifier was published by The Cancer Genome Atlas (TCGA), successfully classifying 74% of primary prostate cancers into seven groups based on one cancer sample per patient. Here, we explore the clinical usefulness of this classification by testing the classifier’s performance in a multifocal context. We analyzed 106 cancer samples from 85 distinct cancer foci within 39 patients. By somatic mutation data from whole-exome sequencing and targeted qualitative and quantitative gene expression assays, 31% of the patients were uniquely classified into one of the seven TCGA classes. Further, different samples from the same focus had conflicting classification in 12% of the foci. In conclusion, the level of both intra- and interfocal heterogeneity is extensive and must be taken into consideration in the development of clinically useful molecular classification of primary prostate cancer.

## Introduction

Prostate cancer is the most common cancer type among men in developed countries, with more than 750,000 new cases worldwide each year. Although some cancers are slow growing, the number of prostate cancer related deaths is high, making prostate cancer the third most common cause of cancer related deaths in men in developed countries^1^.

Prostate cancer patients are classified into risk groups (high, medium or low) based on TNM stage, Gleason score and level of prostate specific antigen (PSA)^2^. Although PSA is highly recognized as a good marker for relapse after prostatectomy, it is far from ideal at the time of diagnosis as aggressive cancers have been reported without a rise in PSA^3^. For several other cancer types, molecular classification predicting prognosis and/or treatment responses are reported. However, clear benefit from such attempts is lacking in prostate cancer^4,5^.

A challenge in the development of such molecular tests for prostate cancer is the multifocal nature of the disease. That is, 60–90% of the patients have multiple distinct cancer foci within the prostate gland at time of diagnosis^6^. The separate cancer foci may have different aggressiveness and develop independently of one another. Heterogeneity in morphology and DNA ploidy has been shown^7^ and a high degree of interfocal heterogeneity of point mutations have recently been reported where the vast majority of cancer foci within the same prostate gland do not share any somatic mutations^8^.

Some molecular biomarker studies have searched for improvements in separating aggressive prostate cancers from indolent ones^9,10^. Others have investigated the overexpression and gene fusions involving four ETS transcription factors^11–13^.

The classifier with most promise was published by The Cancer Genome Atlas (TCGA) in 2015. They reported a molecular taxonomy of primary prostate cancer by applying multiple omics-technologies on 333 primary prostate cancers, including one malignant tissue sample per patient^14^. They identified seven molecular classes combining fusions or overexpression of four ETS transcription factors (*ERG*, *ETV1*, *ETV4*, and *FLI1*) and somatic point mutations in three genes (*SPOP*, *FOXA1*, and *IDH1*). In the study, 74% of the investigated cancer samples were classified based on these events. However, the classification was not correlated to clinical data.

To explore the clinical usefulness of this classification in light of the multifocal nature of prostate cancer, we have investigated how the performance of the TCGA classification is affected by intra-patient heterogeneity in a series of multifocal primary prostate cancer.

## Results

### Molecular classification of multifocal prostate cancer

**Figure 1:**
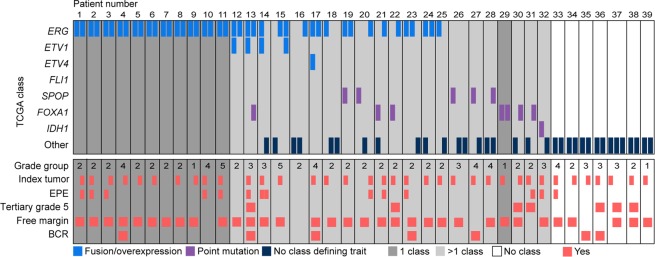
Molecular classes and clinicopathological information per patient and focus. Each column represents one tumor focus, and neighboring bars derive from the same patient. Each row represents the alterations defining a TCGA molecular class (upper panel) or clinicopathological feature (lower panel) Dark grey background: all samples from the patient have the same molecular class; light grey background: samples from the patient correspond to more than one molecular class; white background: none of the samples from the patient can be assigned to any of the seven TCGA classes. EPE: extra prostatic extension, BCR: biochemical recurrence.

We successfully classified 58% (61/106) of the cancer samples into one of the TCGA classes (Supplementary Table S3). In 2 out of these 106, we saw both overexpression/fusion and somatic point mutations. In total, 52% (44/85) of all tumor foci could be classified into one class, 36% (31/85) of the foci did not harbor any of the traits included in the TCGA classification, and 12% (10/85) had conflicting traits (Fig. 1). 20 out of 39 patients had molecular tumor alterations summarized into more than one of the defined classes, 12 patients were defined by a single class and in 7 patients none of the TCGA defined tumor traits were detected.

To make a suitable comparison to TCGA (in which 74% of the patients/samples were assigned to one of the molecular classes), one sample was randomly selected from each of the available 36 index tumors (Fig. 1). In this setting, 25/36 (69%) were successfully classified into a single class.

### Prevalence of fusion transcripts including *ERG*

**Figure 2:**
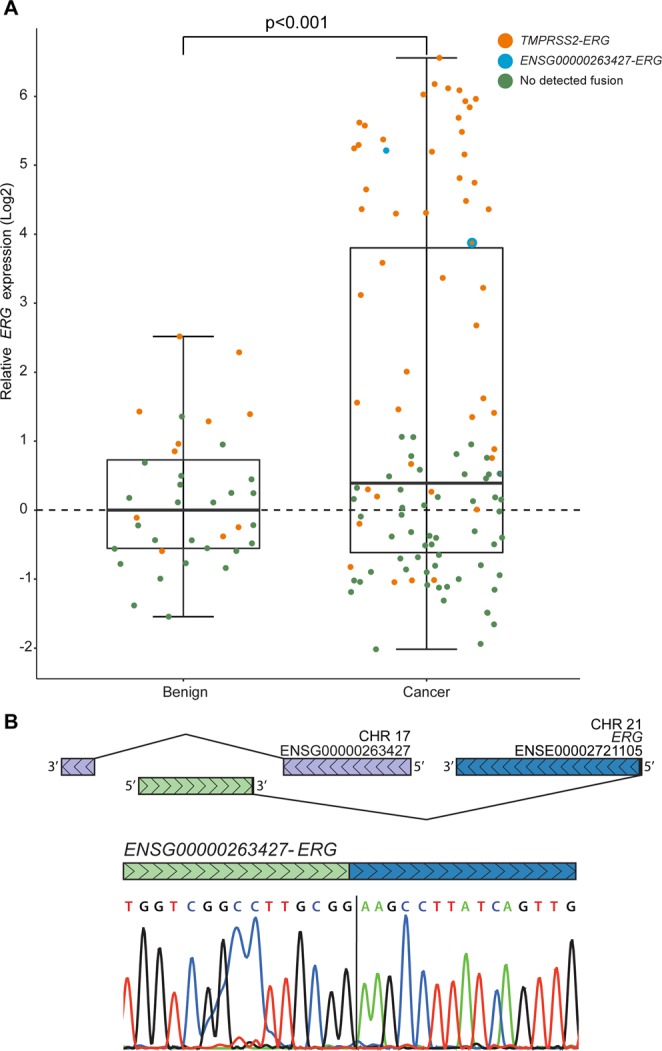
*ERG* expression and validation of a novel fusion transcript. (**A**) Expression levels of 3′-part of *ERG* and presence of *TMPRSS2-ERG* in individual prostate cancer and benign tissue samples. Orange: *TMPRSS2-ERG*, Blue: *ENSG00000263427-ERG*, Green: no detected fusion. (**B**) Identification of a novel fusion transcript consisting of a region antisense to the long non-coding RNA ENSG00000263427 (Ensembl GRCh38; Chr. 17: 8,057,149) and exon 2 of *ERG* (ENSE00003712731; Chr. 21: 38,445,621).

We confirmed the expected high prevalence of *TMPRSS2*-*ERG* fusions in the cohort (60/145 samples (41%); Fig. 2A and Supplementary Fig. S1). Fusion positive samples showed variable expression levels of *ERG* (measured with a real-time RT-PCR; Fig. 2A and Supplementary Table S2). In total, 51% (43/85) of the cancer foci, representing 64% of the patients, had at least one *TMPRSS2-ERG* positive sample. In the benign sample material, 28% (11/39) had a detectable *TMPRSS2-ERG* fusion transcript. Interestingly, one cancer sample had a high expression of *ERG* without any detectable *TMPRSS2-ERG* transcript (Fig. 2A). In this particular sample, analysis of an unpublished RNA sequencing dataset revealed a novel fusion transcript consisting of a region antisense to the long non-coding RNA *ENSG00000263427* (Ensembl GRCh38) fused to exon 2 of *ERG* (ENSE00003712731). The fusion transcript was validated with RT-PCR and Sanger Sequencing (Fig. 2B and Supplementary Fig. S2). The new fusion transcript was also validated in another sample from the same cancer focus. The expression of *ERG* was significantly higher in cancer samples compared to benign samples (P < 0.001). Also, the expression of *ERG* in cancer samples with *TMPRSS2-ERG* was significantly higher than in cancer samples without the fusion transcript (P < 0.001). In comparison, this was not significant in the benign samples (P = 0.1).

### Expression levels of ETS transcription factors

**Figure 3:**
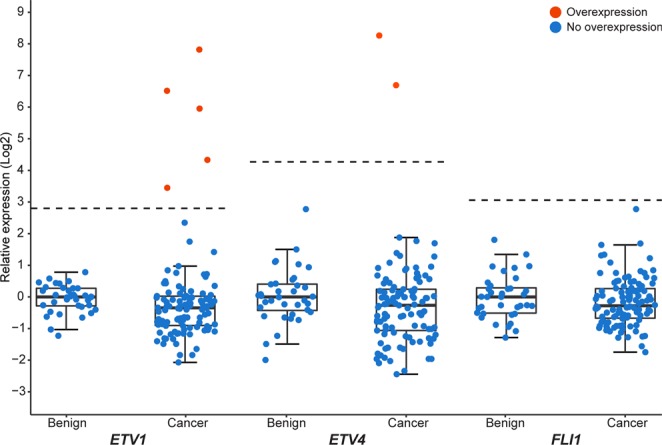
Expression level of three ETS transcription factors in prostate cancer and benign samples. The expression values for each of *ETV1*, *ETV4*, and *FLI1* are relative to the median among the benign samples on a log-2 scale. Thresholds for overexpression are indicated by the dashed lines. Orange: sample with overexpression of the ETS factor; blue: sample without overexpression of the ETS factor.

Semi-quantitative gene expression analysis was also performed on *ETV1*, *ETV4*, and *FLI1* (Fig. 3). This demonstrated that the 3′-end of *ETV1* was overexpressed in 5% (5/106) of the tumor samples. These samples were from one cancer focus in each of four patients. In the two samples with highest *ETV1* expression, RNA-sequencing and validation with RT-PCR revealed a fusion transcript containing *TMPRSS2* and *ETV1* (Supplementary Table S3, transcript validation; Supplementary Fig. S3). *ETV4* overexpression was found in two of the 106 cancer samples (Fig. 3), both deriving from the same cancer focus. Lastly, *FLI1* was not found to be overexpressed in any of the included samples (Fig. 3). None of these genes were overexpressed in any of the benign samples.

### Somatic mutations of *SPOP*, *FOXA1* and *IDH*

Somatic point mutations in *SPOP* were found in six cancer samples from five foci in five patients. Eight samples from seven foci in six patients had point mutations in *FOXA1*. The same mutation in *IDH1* was identified in two samples from one focus within a single patient (Supplementary Table S3). All mutations were validated by Sanger sequencing (see Supplementary Fig. S4 for example results).

### Correlation between focal molecular class and clinicopathological parameters

We found no evidence for correlation between the TCGA molecular class of the different foci and any of the clincopathological parameters grade group, extra prostatic extension, presence of tertiary grade 5, free margins after surgery, or biochemical recurrence (Fig. 1). Seven patients have experienced biochemical recurrence since their surgery (median follow-up = 73 months), but we observed no evidence for correlation between recurrence and molecular class in these patients.

## Discussion

Molecular classification of prostate cancer is highly warranted to aid in the prognostication and treatment selection for this patient group. A promising such classification was proposed by the TCGA in 2015^14^. However, as shown here, the clinical usefulness of this classification is greatly challenged by inter- and intrafocal heterogeneity, reducing the number of uniquely classified patients from 74% in the TCGA study, to 31% in our multifocal cohort.

On the sample level, the unique classifications were more similar to TCGA, with 58% belonging to a single class. Although we see some samples with multiple molecular traits, most samples either lack or have one class defining alteration.

However, high levels of both inter- and intrafocal heterogeneity was observed. Patients were often found to have more than one ETS-factor aberration (13%, 5/39) or at least one focus without any of the molecular traits in the TCGA classification (56%, 22/39). One recent study including patients from 5 different cohorts (total number of patients = 64) are in agreement with the present results, although it is not clear whether the samples were from distinct tumor foci^15^.

The fraction of prostate cancer samples harboring a *TMPRSS2-ERG* fusion transcript found in this study was in concordance with previously published results. Fusion genes are known to impact the measured expression of downstream partner genes due to coupling of a strong promoter to an otherwise weakly expressed gene^11,16^. Therefore, it is not surprising that 30 of the 31 samples with highest expression of *ERG* harbored the *TMPRSS2-ERG* fusion transcript. This was underscored by the observed significant difference in *ERG* expression between the fusion positive and negative cancer samples. Because of the high sensitivity of the RT-PCR, the fusion transcript might be detected although only present in few copies and without overexpression of *ERG*, as seen both in benign and cancer samples. Areas that appear as benign under the microscope could therefore possibly contain a minute amount of cells that express *TMPRSS2-ERG*, but not enough to classify the bulk sample as overexpressing *ERG*. A possible mechanism for the presence of fusion transcript across all expression levels of *ERG* was recently reported, as chimeric transcripts present in normal cells might guide genomic rearrangements on the genomic level leading to oncogenic fusion genes. In that same study, fusion transcripts are also reported in samples from benign tissues^17^.

To our knowledge, this study is the first description of the *ENSG00000263427-ERG* fusion transcript (Fig. 2B and Supplementary Fig. S2). Interestingly, this genomic region contains a predicted binding motif for the transcription factor *FOXA1* (ENSR00000090941, Ensembl, GrCh38), which is frequently mutated in prostate cancer^14^.

The percentage of samples overexpressing *ETV1* (5%) was as expected from previous reports on primary prostate cancer^12,14^. *ETV1* is a known fusion partner in prostate cancer with upstream genes like *SLC45A3*^11^. The validated fusion with *TMPRSS2* in two samples in our cohort indicates a possible mechanism for overexpression of *ETV1*. *ETV4* is reported to be overexpressed in 2–4% of prostate cancers^12,14^, and this is similar to the results in our cohort. *FLI1* is overexpressed in approximately 1% of samples as shown in larger cohorts^12,14^. Therefore, it is not surprising that we find no overexpression of *FLI*.

The present results confirm the low frequencies of somatic point mutations in *FOXA1*, *SPOP* and *IDH1* in prostate cancers^14,18,19^. Mutations in *SPOP* and *FOXA1* have been reported as mutually exclusive from ETS overexpression and fusion events^14^. However, we observed two cancer samples with both *SPOP* or *FOXA1* mutations and fusion of an ETS transcription factor (Supplementary Table S3). A possible explanation for this could be the collision of two tumor foci or the existence of multiple cancer subclones with different genetic features, thus combining these in the sampling of tissue. As we do not have data on the single-cell level, we cannot ascertain whether the point mutations co-occur with the *ETS* fusion in the same cancer cells. It could also be that the mutations found in these two samples are passenger mutations, co-occurring with the ETS overexpression or fusion.

The most clinically relevant finding is that the molecular classification will differ when more than one cancer focus is tested. However, four of the seven patients with biochemical relapse, had expression of *TMPRSS2-ERG* in two separate foci. The role of this fusion in aggressive cancer is, however, debated^11^. *ETV1*, overexpressed in one of the relapsed patients, has previously been linked to abnormal growth *in vitro*^20^ and knockdown of *ETV1* showed significantly reduced invasion in the same study. The ETS factor *ETV4*, found to be overexpressed in one patient within the present series, has been proposed as a marker of more aggressive metastatic prostate cancer^20^. Any other potential clinicopathological relevance of the TCGA classification remains unknown.

In conclusion, the intra- and inter-focal molecular heterogeneity of prostate cancer challenge the clinical usefulness of the proposed molecular classification, as the majority of both cancer foci and patients are classified into multiple classes.

## Methods

### Patient cohort and fresh-frozen tissue samples

The total cohort consisted of 571 prostate cancer patients who underwent prostatectomies between 2010 and 2012 at Oslo University Hospital-*Radiumhospitalet*. Ethical approval for the study was obtained from the regional Ethics Committee South-East Norway (2013/595). All included patients had acinar adenocarcinomas and informed consent was obtained from all patients before initiation of the study. All methods were performed according to the relevant guidelines and regulations. From this cohort, 39 patients with fresh-frozen cancer samples from at least two clearly distinct cancer foci were selected for this study. From each patient, samples from two or three distinct cancer foci and one corresponding benign tissue sample were included. In total, the sample set consisted of 145 cancer and benign samples (106 and 39, respectively) representing 85 distinct cancer foci.

From each prostatectomy, three to seven tissue cores with diameter of 6 mm were biobanked (frozen). The holes were marked and the sections and orientation photographed. Both cancer and benign samples were selected by a pathologist, based on microscopy of hematoxylin and eosin stained sections through formalin-fixed and paraffin-embedded tissue blocks of the remaining tissue. Samples were considered benign if they had no tumor tissue present around the site of the sample used for DNA and RNA extraction.

Total RNA and DNA were isolated from all samples using the AllPrep DNA/RNA/miRNA Universal Kit (Qiagen, Venlo, Netherlands) according to the manufacturer’s protocol.

### Semi-quantitative gene expression of ETS transcription factors

Gene expression levels were investigated with real-time reverse transcription (RT) polymerase chain reaction (PCR). cDNA was made from total RNA using either the High Capacity cDNA Reverse Transcription Kit (Thermo Fisher Scientific, Waltham, MA, USA) or SMARTer™ RACE cDNA Amplification kit (Clontech, Mountain View, CA, USA) according to the manufacturers’ protocols. Expression of four ETS factors (*ERG*, *ETV1*, *ETV4*, and *FLI1*) and one endogenous control (*ABL1*) were then analysed using TaqMan gene expression assays (Thermo Fisher Scientific.) Assay IDs were as following: Hs01554630_m1, targeting exons 6 and 7 of *ERG* (ENSE00003536380 to ENSE00003462848). Hs00231877_m1, targeting exons 7 and 8 of *ETV1* (ENSE00003595382 to ENSE00003600886). Hs00944562_m1, targeting exons 5 and 6 of *ETV4* (ENSE00003471192 to ENSE00003665992). Hs00956709_m1, targeting exons 5 and 6 of *FLI1* (ENSE00003711049 and ENSE00003702967). Hs01104728_m1, targeting exons 8 and 9 of *ABL1* (ENSE00001741732 and ENSE00001715343). All assays target exon-exon junctions downstream of common fusion breakpoints, measuring a combination of wild-type ETS - and fusion transcripts. All assays were run in triplicates on ABI 7900HT Fast Real-time PCR System (Applied Biosystems, Foster City, CA, USA).

### Fusion gene expression

Detection of the fusion transcripts *TMPRSS2-ERG* and *AC129492*.*2-201-ERG* was performed with RT-PCR on the previously described cDNA from all samples using the HotStar Taq DNA polymerase Kit (Qiagen) according to the manufacturer’s protocol. Primers were designed using the Primer3 software (Supplementary Table S1; http://bioinfo.ut.ee/primer3-0.4.0/). Primers were designed to pick up multiple *TMPRSS2-ERG* fusion transcript variants. Visualization of RT-PCR products with gel electrophoresis was performed with a 2% agarose gel and 200 V for 30 minutes. RT-PCR and UV-visualization were performed at least twice for all samples. For samples with inconclusive results after two rounds of RT-PCR and UV-visualization, an additional RT-PCR reaction and agarose gel was run for verification.

### Detection of somatic point mutations

All 145 tissue samples have been previously analyzed by whole-exome sequencing, and the somatic mutation data have been published elsewhere^8^. Sequence variants from three different genes (*SPOP*, *FOXA1* and *IDH1*) were nominated from the exome sequencing analysis and here validated on genomic DNA with PCR and Sanger sequencing (using the same set up, kit, and cycling conditions as the RT-PCR described previously). The Primer3 software was used for primer design (Supplementary Table S1). Sanger-sequencing was performed on a MJ tetrad (BIO-RAD, Ca, USA) and an AB3730 DNA analyzer (Applied Biosystems) according to the manufacturers’ protocols.

### Statistical analyses

Cycle threshold (CT) values were recorded for all gene expression assays (Supplementary Table S2), and this gene expression was normalized using the endogenous control *ABL1* ($\Delta$ *CT* = *CT*_*analyzed gene*_ *− CT*_*endogenous control*_). *ETV1*, *ETV4*, and *FLI1* were considered overexpressed in a sample if the expression was higher than $Q3+3\bullet IQR$ (Q3; 75 percentile of Δ*CT-*values across all samples, IQR; interquartile range of $\Delta$*CT*-values across all samples). All statistical comparisons between groups were performed by 2-sided paired sample t-tests, using the “t.test()” function in R with “alternative = two.sided” and “paired = TRUE”.

### Molecular classification

The seven molecular classes from TCGA are defined by either overexpression or fusion of one of four ETS transcription factors (*ERG*, *ETV1*, *ETV4*, or *FLI1*) or by somatic point mutations in one of three genes (*SPOP*, *FOXA1*, or *IDH1*). A sample was classified into a single class if it held one of the seven investigated features. If all investigated samples from the same focus held one of the investigated features, the focus was assigned to a single molecular class. If all investigated samples from one patient had only one class-defining molecular trait, this patient was classified into a single molecular class. If multiple defining molecular traits were identified, the sample/focus/patient was assigned to more than one molecular class.

## Supplementary information

Supplementary information

## Data Availability

The datasets generated during and/or analysed during the current study are available from the corresponding author on reasonable request.
