# Supplementary material for: Interfocal heterogeneity challenges the clinical usefulness of molecular classification of primary prostate cancer
Source: Sci Rep. 2019 Sep 19;9:13579. doi: 10.1038/s41598-019-49964-7 (PMC6753093; doi:10.1038/s41598-019-49964-7)
Supplement: Supplementary file 1 — Supplementary information [file 41598_2019_49964_MOESM1_ESM.pdf]

Interfocal heterogeneity challenges the clinical usefulness of molecular classification of primary prostate cancer

Kristina Totland Carm[1,2], Andreas M. Hoff[1], Anne Cathrine Bakken[1], Ulrika Axcrona[3], Karol Axcrona[4], Ragnhild A. Lothe[1,2], Rolf I. Skotheim[1,5,*], and Marthe Løvf[1]

SUPPLEMENTARY INFORMATION

Supplementary Table S1 - Oligonucleotides used as primers in the PCR assays

Gene	Name	Type	Length	Sequence	Melting temperature	GC content (%)
FOXA1	FOXA1_EX4EARLY_F	Forward	21	CAACAACCTCATGTCCTCCTC	59.6	52.4
FOXA1	FOXA1_EX4EARLY_R	Reverse	20	ATGGCTATGCCAGACAAACC	59.6	50.0
FOXA1	FOXA1_EX4LATE_F	Forward	20	CCGCCCTACTCGTACATCTC	59.7	60.0
FOXA1	FOXA1_EX4LATE_R	Reverse	20	GGAGGCTGGAGTCTTCAACT	58.5	55.0
SPOP	SPOP_INTEX11_F	Forward	20	TGTTTTGGACAGGTGTTTGC	59.6	45.0
SPOP	SPOP_INTEX12_R	Reverse	21	CTCATCAGATCTGGGAACTGC	59.8	52.4
IDH1	IDH1_EX7_F	Forward	22	TGAAACAAATGTGGAAATCACC	59.7	36.4
IDH1	IDH1_EX7_R	Reverse	23	TTCATACCTTGCTTAATGGGTGT	59.8	39.1
TMPRSS2	TMPRSS2_ex1_F	Forward	18	GGGGAGCGCCGCCTGGAG	70.4	83.3
ERG	ERG_ex4_R	Reverse	21	CCCACCATCTTCCCGCCTTTG	68.2	61.9
AC129492.2	RP11599B13_F	Forward	20	AGGCAGGTTCCCCTACATCT	62.0	55.0
ERG	ERG_ex2_R	Reverse	20	AACAACGACTGGTCCTCACT	60.0	50.0

Supplementary Table S2 - Ct values - TaqMan gene expression assay

Sample	Cancer/ Benign	*ABL1* median Ct	*ERG* median Ct	Delta Ct (Ct*ERG* - Ct*ABL1*)	*ETV1* median Ct	Delta Ct (Ct*ETV1*- Ct*ABL1*)	*ETV4* median CT	Delta Ct (Ct*ETV4* - Ct*ABL1*)	*FLI1* median CT	Delta Ct (Ct*FLI1* - Ct*ABL1*)
Patient B01 BS	B	26.86	31.38	4.52	29.17	2.31	31.52	4.66	28.47	1.61
Patient B01 S1 F1	C	27.58	31.59	4.01	30.24	2.66	32.88	5.29	29.78	2.19
Patient B01 S2 F1	C	27.21	28.32	1.11	29.58	2.37	32.95	5.75	30.00	2.79
Patient B01 S3 F2	C	26.68	29.47	2.79	30.06	3.38	32.71	6.03	30.19	3.52
Patient B02 BS	B	26.63	32.94	6.31	29.28	2.65	31.80	5.17	31.33	4.70
Patient B02 S1 F1	C	26.30	25.66	-0.64	30.44	4.13	33.00	6.69	31.01	4.71
Patient B02 S2 F1	C	26.68	26.78	0.10	30.19	3.51	32.68	6.00	30.40	3.72
Patient B02 S3 F2	C	26.88	31.60	4.72	29.94	3.06	32.46	5.58	30.16	3.28
Patient B03 BS	B	26.74	29.69	2.95	30.14	3.40	33.12	6.38	30.48	3.74
Patient B03 S1 F1	C	27.04	29.15	2.10	30.53	3.49	32.06	5.02	30.47	3.43
Patient B03 S2 F1	C	26.52	26.84	0.32	29.77	3.25	32.10	5.57	29.78	3.26
Patient B03 S3 F2	C	26.99	32.19	5.20	30.21	3.23	32.79	5.80	29.59	2.60
Patient B04 BS	B	28.02	32.13	4.11	30.65	2.63	34.04	6.02	30.47	2.46
Patient B04 S1 F1	C	26.98	32.65	5.67	30.88	3.90	32.95	5.97	30.79	3.81
Patient B04 S2 F2	C	27.29	28.45	1.16	29.95	2.66	32.38	5.09	30.28	2.99
Patient B04 S3 F2	C	26.66	27.65	0.99	30.22	3.56	32.78	6.12	30.48	3.82
Patient B05 BS	B	27.50	32.73	5.22	30.70	3.20	33.09	5.59	30.00	2.50
Patient B05 S1 F1	C	27.07	30.98	3.91	30.81	3.74	32.19	5.12	29.15	2.08
Patient B05 S2 F1	C	27.00	29.36	2.35	30.86	3.85	32.81	5.80	29.66	2.66
Patient B05 S3 F2	C	26.50	27.22	0.72	30.23	3.73	33.23	6.74	30.32	3.82
Patient B06 BS	B	27.45	31.96	4.51	30.48	3.02	32.28	4.83	29.90	2.45
Patient B06 S1 F1	C	27.15	31.96	4.80	31.34	4.18	34.39	7.23	31.47	4.31
Patient B06 S2 F2	C	26.63	26.81	0.18	30.60	3.97	34.43	7.80	31.14	4.51
Patient B07 BS	B	28.10	33.20	5.10	31.15	3.05	32.84	4.74	30.17	2.07
Patient B07 S1 F1	C	26.67	27.32	0.66	29.70	3.03	32.33	5.67	30.29	3.62
Patient B07 S2 F2	C	27.50	28.67	1.17	30.71	3.21	32.96	5.46	30.16	2.66
Patient B08 BS	B	26.77	29.96	3.18	29.95	3.17	32.70	5.93	30.47	3.70
Patient B08 S1 F2	C	27.06	26.85	-0.22	31.28	4.22	34.07	7.01	31.13	4.07
Patient B08 S2 F1	C	26.92	26.46	-0.46	30.96	4.04	33.20	6.28	30.47	3.55
Patient B09 BS	B	27.03	31.65	4.62	30.75	3.72	32.91	5.88	30.98	3.95
Patient B09 S1 F1	C	27.33	27.60	0.28	30.55	3.22	33.24	5.91	31.24	3.91
Patient B09 S2 F2	C	27.21	26.84	-0.37	31.49	4.27	33.60	6.39	31.86	4.65
Patient B10 BS	B	26.65	31.67	5.02	29.25	2.60	30.92	4.27	29.61	2.96
Patient B10 S1 F1	C	25.93	26.19	0.26	29.27	3.34	32.71	6.78	30.28	4.35
Patient B10 S2 F1	C	26.44	28.03	1.60	29.18	2.74	31.53	5.09	29.24	2.81
Patient B10 S3 F2	C	26.89	26.74	-0.15	29.01	2.12	32.10	5.21	29.97	3.08
Patient B11 BS	B	26.90	32.97	6.06	30.26	3.35	32.31	5.41	30.86	3.95
Patient B11 S1 F1	C	27.32	26.83	-0.49	29.57	2.25	34.40	7.07	30.24	2.92
Patient B11 S2 F1	C	27.26	26.70	-0.55	28.93	1.67	33.01	5.75	29.59	2.33
Patient B11 S3 F2	C	26.55	26.54	-0.01	30.55	4.00	33.96	7.41	30.31	3.77
Patient B12 BS	B	27.51	32.87	5.36	30.77	3.26	32.88	5.37	30.11	2.60
Patient B12 S1 F1	C	27.32	33.81	6.48	26.97	-0.36	34.11	6.79	31.66	4.34
Patient B12 S2 F2	C	27.17	29.42	2.25	30.40	3.23	32.96	5.79	30.65	3.48
Patient B13 BS	B	26.89	32.92	6.03	29.98	3.08	32.24	5.35	30.96	4.07
Patient B13 S1 F1	C	26.30	31.01	4.71	21.57	-4.73	31.64	5.34	29.97	3.67
Patient B13 S2 F1	C	26.55	30.02	3.46	23.13	-3.42	31.43	4.87	29.99	3.44
Patient B13 S3 F2	C	26.15	32.45	6.29	29.60	3.44	30.05	3.89	30.63	4.47
Patient B14 BS	B	26.30	32.36	6.06	29.12	2.82	31.99	5.68	29.98	3.68
Patient B14 S1 F1	C	26.71	31.39	4.69	25.47	-1.24	32.45	5.74	29.67	2.97
Patient B14 S2 F1	C	26.72	30.84	4.12	28.06	1.34	31.89	5.18	29.94	3.23
Patient B14 S3 F2	C	26.73	31.90	5.17	29.06	2.33	31.20	4.47	29.96	3.23
Patient B15 BS	B	27.58	32.87	5.29	30.95	3.37	32.80	5.22	30.64	3.06
Patient B15 S1 F1	C	27.59	34.17	6.58	32.52	4.93	35.32	7.73	31.09	3.50
Patient B15 S2 F2	C	28.11	33.28	5.17	32.36	4.25	34.92	6.81	31.62	3.51
Patient B15 S3 F3	C	27.82	34.31	6.49	24.96	-2.86	35.44	7.62	32.52	4.69
Patient B16 BS	B	27.02	34.04	7.01	31.14	4.12	34.28	7.26	31.52	4.50
Patient B16 S1 F1	C	26.95	33.90	6.96	31.10	4.15	34.13	7.18	31.35	4.40
Patient B16 S2 F2	C	27.28	34.77	7.49	28.03	0.75	34.69	7.40	32.26	4.98
Patient B16 S3 F3	C	27.01	31.60	4.59	30.46	3.45	31.39	4.38	30.39	3.38

Patient B17 BS	B	27.19	33.10	5.91	30.55	3.36	33.37	6.18	31.11	3.92
Patient B17 S1 F1	C	26.96	33.29	6.33	30.12	3.16	24.47	-2.49	30.97	4.01
Patient B17 S2 F1	C	27.36	32.82	5.46	30.65	3.29	26.44	-0.92	31.69	4.33
Patient B17 S3 F2	C	27.12	27.35	0.23	30.29	3.17	33.86	6.73	31.13	4.00
Patient B18 BS	B	27.11	32.80	5.69	30.76	3.64	32.89	5.78	30.53	3.42
Patient B18 S1 F3	C	27.86	34.13	6.27	32.79	4.93	35.59	7.73	32.26	4.40
Patient B18 S2 F1	C	27.23	28.34	1.11	31.97	4.74	33.79	6.56	27.87	0.64
Patient B18 S3 F2	C	27.02	33.19	6.17	30.42	3.40	33.40	6.38	30.95	3.92
Patient B19 BS	B	27.40	33.09	5.69	30.89	3.50	34.40	7.00	31.17	3.78
Patient B19 S1 F1	C	27.58	34.09	6.51	32.16	4.58	35.79	8.21	31.56	3.98
Patient B19 S2 F2	C	27.04	26.93	-0.10	30.82	3.78	34.77	7.73	30.96	3.92
Patient B20 BS	B	26.63	30.81	4.18	29.73	3.10	32.87	6.25	29.86	3.23
Patient B20 S1 F1	C	26.66	31.60	4.94	30.05	3.39	32.59	5.93	29.81	3.14
Patient B20 S2 F2	C	26.72	32.21	5.49	30.19	3.47	33.07	6.34	30.60	3.88
Patient B20 S3 F3	C	26.90	30.75	3.85	29.88	2.98	32.45	5.56	30.29	3.39
Patient B21 BS	B	26.71	30.75	4.04	29.50	2.78	32.47	5.76	30.05	3.34
Patient B21 S1 F1	C	26.98	32.86	5.87	31.26	4.28	33.88	6.90	31.19	4.21
Patient B21 S2 F2	C	26.81	28.69	1.89	30.60	3.80	32.44	5.63	30.63	3.82
Patient B21 S3 F1	C	26.91	32.06	5.15	30.98	4.07	33.47	6.55	30.48	3.57
Patient B22 BS	B	27.55	34.41	6.85	30.45	2.90	33.65	6.10	31.92	4.36
Patient B22 S1 F1	C	26.89	33.36	6.47	31.15	4.25	33.84	6.95	31.24	4.35
Patient B22 S2 F2	C	27.68	32.95	5.27	30.44	2.76	33.21	5.53	30.90	3.23
Patient B23 BS	B	26.86	31.65	4.78	29.89	3.03	29.86	2.99	30.01	3.15
Patient B23 S1 F1	C	26.59	25.88	-0.71	29.57	2.98	32.60	6.01	29.99	3.40
Patient B23 S2 F2	C	26.64	30.70	4.06	30.41	3.77	32.44	5.80	30.69	4.05
Patient B23 S3 F3	C	26.64	31.30	4.66	29.75	3.11	30.72	4.07	30.22	3.58
Patient B24 BS	B	27.79	33.64	5.85	30.60	2.81	33.39	5.59	31.20	3.41
Patient B24 S1 F1	C	27.99	33.95	5.97	31.30	3.31	34.21	6.22	31.36	3.38
Patient B24 S2 F1	C	27.00	27.82	0.82	30.87	3.88	33.32	6.32	30.75	3.75
Patient B24 S3 F2	C	26.76	25.67	-1.09	29.94	3.18	33.13	6.37	31.30	4.54
Patient B25 BS	B	27.99	33.71	5.72	31.74	3.75	33.97	5.98	31.67	3.68
Patient B25 S1 F2	C	28.53	32.94	4.41	31.58	3.06	33.74	5.21	30.30	1.77
Patient B25 S2 F1	C	27.43	26.81	-0.61	30.92	3.49	32.95	5.52	30.21	2.78
Patient B25 S3 F2	C	26.73	32.16	5.43	30.62	3.89	33.09	6.37	28.83	2.11
Patient B26 BS	B	27.02	32.38	5.36	30.63	3.60	33.52	6.50	30.30	3.28
Patient B26 S1 F1	C	26.91	32.89	5.98	30.79	3.88	33.61	6.70	30.70	3.79
Patient B26 S2 F2	C	27.07	34.03	6.96	31.56	4.49	34.16	7.09	31.46	4.39
Patient B26 S3 F3	C	27.39	33.17	5.78	30.24	2.84	33.19	5.80	30.32	2.92
Patient B27 S1 F1	C	27.56	33.68	6.12	31.37	3.81	33.62	6.06	31.09	3.53
Patient B27 S2 F1	C	27.88	33.28	5.40	30.99	3.11	33.68	5.81	30.88	3.01
Patient B27 S3 F2	C	28.21	33.75	5.54	31.16	2.95	33.06	4.85	31.46	3.25
Patient B28 BS	B	26.99	33.45	6.46	31.31	4.32	34.75	7.76	31.29	4.30
Patient B28 S1 F1	C	26.73	34.14	7.41	31.89	5.16	34.84	8.11	31.89	5.16
Patient B28 S2 F2	C	26.99	33.49	6.49	31.40	4.40	34.19	7.20	31.50	4.51
Patient B28 S3 F2	C	27.09	33.36	6.28	31.66	4.58	34.05	6.96	30.99	3.90
Patient B29 BS	B	26.77	31.75	4.97	30.34	3.56	31.41	4.64	29.60	2.83
Patient B29 S1 F1	C	26.78	33.91	7.13	30.92	4.14	33.29	6.51	30.92	4.14
Patient B29 S2 F2	C	26.88	33.48	6.59	30.25	3.37	33.14	6.25	30.67	3.79
Patient B30 S1 F1	C	26.50	31.38	4.89	29.58	3.08	32.59	6.10	29.63	3.14
Patient B30 S2 F2	C	26.75	32.09	5.34	30.09	3.34	32.33	5.58	29.69	2.95
Patient B31 BS	B	26.97	32.99	6.02	30.04	3.08	33.29	6.33	31.23	4.27
Patient B31 S1 F2	C	27.70	33.49	5.79	31.72	4.02	35.08	7.37	31.74	4.04
Patient B31 S2 F1	C	26.96	32.27	5.31	30.22	3.26	32.57	5.61	30.70	3.74
Patient B31 S3 F2	C	27.09	32.37	5.28	31.05	3.96	34.46	7.37	31.40	4.31
Patient B32 BS	B	26.90	30.98	4.08	29.42	2.51	32.37	5.47	30.32	3.42
Patient B32 S1 F1	C	26.97	32.81	5.84	30.25	3.29	31.89	4.92	30.39	3.42
Patient B32 S2 F1	C	26.97	32.94	5.97	30.40	3.43	31.78	4.81	31.06	4.10
Patient B32 S3 F2	C	26.72	31.70	4.98	30.11	3.39	31.41	4.69	29.63	2.91
Patient B33 BS	B	26.45	31.67	5.22	29.50	3.05	32.17	5.72	30.18	3.73
Patient B33 S1 F1	C	26.89	31.40	4.52	30.50	3.61	33.57	6.68	29.97	3.09
Patient B33 S2 F1	C	27.15	32.46	5.32	31.39	4.24	33.60	6.46	30.90	3.75
Patient B33 S3 F2	C	26.59	31.55	4.95	29.05	2.46	31.92	5.32	29.42	2.82
Patient B34 BS	B	28.03	33.98	5.95	30.93	2.90	33.77	5.74	31.34	3.31
Patient B34 S1 F1	C	26.78	32.93	6.15	30.52	3.74	32.77	5.99	30.97	4.19

Patient B34 S2 F2	C	26.71	33.50	6.78	31.13	4.41	34.41	7.69	31.38	4.67
Patient B34 S3 F2	C	26.42	33.04	6.62	30.25	3.82	35.28	8.85	30.64	4.22
Patient B35 S1 F1	C	27.97	33.82	5.85	31.92	3.95	35.02	7.05	31.36	3.39
Patient B35 S2 F1	C	27.37	31.77	4.41	29.75	2.38	32.21	4.84	29.10	1.73
Patient B35 S3 F2	C	27.75	32.70	4.95	30.35	2.60	31.75	4.00	30.33	2.57
Patient B36 BS	B	27.28	33.53	6.25	30.10	2.82	33.17	5.89	30.45	3.17
Patient B36 S1 F1	C	26.77	33.18	6.41	30.97	4.19	34.02	7.25	31.10	4.33
Patient B36 S2 F2	C	27.12	32.99	5.87	31.13	4.01	33.95	6.83	30.31	3.19
Patient B36 S3 F2	C	26.69	33.07	6.37	29.90	3.20	34.26	7.56	30.64	3.95
Patient B37 BS	B	27.37	33.28	5.91	30.48	3.11	33.79	6.42	31.36	3.98
Patient B37 S1 F1	C	27.03	32.31	5.29	30.68	3.65	33.34	6.31	31.06	4.03
Patient B37 S2 F2	C	27.56	33.13	5.56	30.48	2.91	33.34	5.78	31.08	3.52
Patient B37 S3 F3	C	27.51	32.52	5.01	30.63	3.12	32.00	4.49	30.17	2.66
Patient B38 BS	B	27.67	33.91	6.24	30.34	2.67	34.12	6.46	31.37	3.70
Patient B38 S1 F1	C	27.11	33.76	6.66	31.53	4.42	34.97	7.86	31.54	4.43
Patient B38 S2 F2	C	27.44	34.00	6.56	31.51	4.07	34.82	7.37	31.30	3.86
Patient B39 BS	B	27.80	33.38	5.58	30.97	3.17	33.80	6.00	30.97	3.17
Patient B39 S1 F1	C	27.55	34.06	6.51	31.43	3.88	33.33	5.78	31.66	4.11
Patient B39 S2 F2	C	27.60	33.96	6.37	31.03	3.43	33.23	5.64	31.32	3.72

Supplementary Table S3 - concatenated genomic features and gleason grade groups in cancer samples and foci

Patient	Sample	Focus	Sample subtype	Focus subtype	Patient subtype	*TMPRSS2-ERG*	*ENSG00000263427-ERG*	*TMPRSS2-ETV1*	*ETV1* high	*ETV4* high	*FLI1* high	*SPOP* mutation	*FOXA1* mutation	*IDH1* mutation	Grade group patient	Gleason focus
B01	S1	F1	*ERG*	*ERG*	*ERG*	yes	no	no	no	no	no	no	no	no	2	3+3
B01	S2	F1	*ERG*	*ERG*	*ERG*	yes	no	no	no	no	no	no	no	no	2	3+3
B01	S3	F2	*ERG*	*ERG*	*ERG*	yes	no	no	no	no	no	no	no	no	2	3+4
B02	S1	F1	*ERG*	*ERG*	*ERG*	yes	no	no	no	no	no	no	no	no	2	3+4
B02	S2	F1	*ERG*	*ERG*	*ERG*	yes	no	no	no	no	no	no	no	no	2	3+4
B02	S3	F2	*ERG*	*ERG*	*ERG*	yes	no	no	no	no	no	no	no	no	2	3+3
B03	S1	F1	*ERG*	*ERG*	*ERG*	yes	no	no	no	no	no	no	no	no	2	3+4
B03	S2	F1	*ERG*	*ERG*	*ERG*	yes	no	no	no	no	no	no	no	no	2	3+4
B03	S3	F2	*ERG*	*ERG*	*ERG*	yes	no	no	no	no	no	no	no	no	2	3+3
B04	S1	F1	*ERG*	*ERG*	*ERG*	yes	no	no	no	no	no	no	no	no	4	4+4
B04	S2	F2	*ERG*	*ERG*	*ERG*	yes	no	no	no	no	no	no	no	no	4	4+5
B04	S3	F2	*ERG*	*ERG*	*ERG*	yes	no	no	no	no	no	no	no	no	4	4+5
B05	S1	F1	*ERG*	*ERG*	*ERG*	yes	no	no	no	no	no	no	no	no	2	3+3
B05	S2	F1	*ERG*	*ERG*	*ERG*	yes	no	no	no	no	no	no	no	no	2	3+3
B05	S3	F2	*ERG*	*ERG*	*ERG*	yes	no	no	no	no	no	no	no	no	2	3+4
B06	S1	F1	*ERG*	*ERG*	*ERG*	yes	no	no	no	no	no	no	no	no	2	3+3
B06	S2	F2	*ERG*	*ERG*	*ERG*	yes	no	no	no	no	no	no	no	no	2	3+4
B07	S1	F1	*ERG*	*ERG*	*ERG*	yes	no	no	no	no	no	no	no	no	2	3+4
B07	S2	F2	*ERG*	*ERG*	*ERG*	yes	no	no	no	no	no	no	no	no	2	3+4
B08	S1	F2	*ERG*	*ERG*	*ERG*	yes	no	no	no	no	no	no	no	no	2	3+4
B08	S2	F1	*ERG*	*ERG*	*ERG*	yes	no	no	no	no	no	no	no	no	2	3+4
B09	S1	F1	*ERG*	*ERG*	*ERG*	yes	no	no	no	no	no	no	no	no	1	3+3
B09	S2	F2	*ERG*	*ERG*	*ERG*	yes	no	no	no	no	no	no	no	no	1	3+4
B10	S1	F1	*ERG*	*ERG*	*ERG*	no	yes	no	no	no	no	no	no	no	4	4+4
B10	S2	F1	*ERG*	*ERG*	*ERG*	yes	yes	no	no	no	no	no	no	no	4	4+4
B10	S3	F2	*ERG*	*ERG*	*ERG*	yes	no	no	no	no	no	no	no	no	4	4+3
B11	S1	F1	*ERG*	*ERG*	*ERG*	yes	no	no	no	no	no	no	no	no	5	4+5
B11	S2	F1	*ERG*	*ERG*	*ERG*	yes	no	no	no	no	no	no	no	no	5	4+5
B11	S3	F2	*ERG*	*ERG*	*ERG*	yes	no	no	no	no	no	no	no	no	5	4+5
B12	S1	F1	>1	>1	>1	yes	no	no	yes	no	no	no	no	no	2	3+3
B12	S2	F2	*ERG*	*ERG*	>1	yes	no	no	no	no	no	no	no	no	2	3+3
B13	S1	F1	*ETV1*	>1	>1	no	no	yes	yes	no	no	no	no	no	3	4+3+5
B13	S2	F1	>1	>1	>1	yes	no	yes	yes	no	no	no	no	no	3	4+3+5
B13	S3	F2	>1	>1	>1	yes	no	no	no	no	no	no	p.R262C	no	3	3+3
B14	S1	F1	ETV1	>1	>1	no	no	no	yes	no	no	no	no	no	3	4+3
B14	S2	F1	*ERG*	>1	>1	yes	no	no	no	no	no	no	no	no	3	4+3
B14	S3	F2	No subtype	No subtype	>1	no	no	no	no	no	no	no	no	no	3	4+3
B15	S1	F1	No subtype	No subtype	>1	no	no	no	no	no	no	no	no	no	5	3+4
B15	S2	F2	*ERG*	*ERG*	>1	yes	no	no	no	no	no	no	no	no	5	4+5
B15	S3	F3	>1	>1	>1	yes	no	no	yes	no	no	no	no	no	5	3+4
B16	S1	F1	No subtype	No subtype	>1	no	no	no	no	no	no	no	no	no	2	3+4
B16	S2	F2	No subtype	No subtype	>1	no	no	no	no	no	no	no	no	no	2	3+3
B16	S3	F3	*ERG*	*ERG*	>1	yes	no	no	no	no	no	no	no	no	2	3+3
B17	S1	F1	*ETV4*	>1	>1	no	no	no	no	yes	no	no	no	no	4	3+5
B17	S2	F1	>1	>1	>1	yes	no	no	no	yes	no	no	no	no	4	3+5
B17	S3	F2	*ERG*	*ERG*	>1	yes	no	no	no	no	no	no	no	no	4	3+4
B18	S1	F3	No subtype	No subtype	>1	no	no	no	no	no	no	no	no	no	2	3+4
B18	S2	F1	*ERG*	*ERG*	>1	yes	no	no	no	no	no	no	no	no	2	3+4
B18	S3	F2	No subtype	No subtype	>1	no	no	no	no	no	no	no	no	no	2	3+3
B19	S1	F1	>1	>1	>1	yes	no	no	no	no	no	p.D130V	no	no	2	3+4
B19	S2	F2	*ERG*	*ERG*	>1	yes	no	no	no	no	no	no	no	no	2	3+4
B20	S1	F1	*SPOP*	*SPOP*	>1	no	no	no	no	no	no	p.W131G	no	no	2	3+4
B20	S2	F2	No subtype	No subtype	>1	no	no	no	no	no	no	no	no	no	2	3+3
B20	S3	F3	*ERG*	*ERG*	>1	yes	no	no	no	no	no	no	no	no	2	3+4
B21	S1	F1	No subtype	>1	>1	no	no	no	no	no	no	no	no	no	2	3+4
B21	S2	F2	*ERG*	*ERG*	>1	yes	no	no	no	no	no	no	no	no	2	3+4
B21	S3	F1	*FOXA1*	>1	>1	no	no	no	no	no	no	no	p.R262C	no	2	3+4
B22	S1	F1	*FOXA1*	*FOXA1*	>1	no	no	no	no	no	no	no	p.R233_D236del	no	2	3+4+5
B22	S2	F2	*ERG*	*ERG*	>1	yes	no	no	no	no	no	no	no	no	2	3+3
B23	S1	F1	*ERG*	*ERG*	>1	yes	no	no	no	no	no	no	no	no	2	3+4
B23	S2	F2	*ERG*	*ERG*	>1	yes	no	no	no	no	no	no	no	no	2	3+4
B23	S3	F3	No subtype	No subtype	>1	no	no	no	no	no	no	no	no	no	2	3+3
B24	S1	F1	No subtype	>1	>1	no	no	no	no	no	no	no	no	no	2	3+4
B24	S2	F1	*ERG*	>1	>1	yes	no	no	no	no	no	no	no	no	2	3+4
B24	S3	F2	*ERG*	*ERG*	>1	yes	no	no	no	no	no	no	no	no	2	4+3
B25	S1	F2	No subtype	No subtype	>1	no	no	no	no	no	no	no	no	no	2	3+4
B25	S2	F1	*ERG*	*ERG*	>1	yes	no	no	no	no	no	no	no	no	2	3+4
B25	S3	F2	No subtype	No subtype	>1	no	no	no	no	no	no	no	no	no	2	3+4
B26	S1	F1	*SPOP*	*SPOP*	>1	no	no	no	no	no	no	p.F133V	no	no	3	4+3
B26	S2	F2	No subtype	No subtype	>1	no	no	no	no	no	no	no	no	no	3	3+4
B26	S3	F3	No subtype	No subtype	>1	no	no	no	no	no	no	no	no	no	3	4+3
B27	S1	F1	*SPOP*	*SPOP*	>1	no	no	no	no	no	no	p.F125I	no	no	4	4+4
B27	S2	F1	*SPOP*	*SPOP*	>1	no	no	no	no	no	no	p.F125I	no	no	4	4+4
B27	S3	F2	No subtype	No subtype	>1	no	no	no	no	no	no	no	no	no	4	3+4
B28	S1	F1	No subtype	No subtype	>1	no	no	no	no	no	no	no	no	no	4	5+3
B28	S2	F2	No subtype	>1	>1	no	no	no	no	no	no	no	no	no	4	3+4
B28	S3	F2	*SPOP*	>1	>1	no	no	no	no	no	no	p.F102V	no	no	4	3+4
B29	S1	F1	*FOXA1*	*FOXA1*	*FOXA1*	no	no	no	no	no	no	no	p.H220Q	no	1	3+3
B29	S2	F2	*FOXA1*	*FOXA1*	*FOXA1*	no	no	no	no	no	no	no	p.H220Q	no	1	3+3
B30	S1	F1	No subtype	No subtype	>1	no	no	no	no	no	no	no	no	no	2	3+3
B30	S2	F2	*FOXA1*	*FOXA1*	>1	no	no	no	no	no	no	no	p.S217F	no	2	3+4+5
B31	S1	F2	*FOXA1*	*FOXA1*	>1	no	no	no	no	no	no	no	p.S443Hfs*112, p.S304R	no	2	3+4
B31	S2	F1	No subtype	No subtype	>1	no	no	no	no	no	no	no	no	no	2	3+4+5
B31	S3	F2	*FOXA1*	*FOXA1*	>1	no	no	no	no	no	no	no	p.S443Hfs*112	no	2	3+4
B32	S1	F1	*IDH1*	*IDH1*	>1	no	no	no	no	no	no	no	no	p.R132G	3	4+3
B32	S2	F1	*IDH1*	*IDH1*	>1	no	no	no	no	no	no	no	no	p.R132G	3	4+3
B32	S3	F2	No subtype	No subtype	>1	no	no	no	no	no	no	no	no	no	3	3+3
B33	S1	F1	No subtype	No subtype	No subtype	no	no	no	no	no	no	no	no	no	4	4+4
B33	S2	F1	No subtype	No subtype	No subtype	no	no	no	no	no	no	no	no	no	4	4+4
B33	S3	F2	No subtype	No subtype	No subtype	no	no	no	no	no	no	no	no	no	4	3+4
B34	S1	F1	No subtype	No subtype	No subtype	no	no	no	no	no	no	no	no	no	2	3+3
B34	S2	F2	No subtype	No subtype	No subtype	no	no	no	no	no	no	no	no	no	2	3+4
B34	S3	F2	No subtype	No subtype	No subtype	no	no	no	no	no	no	no	no	no	2	3+4
B35	S1	F1	No subtype	No subtype	No subtype	no	no	no	no	no	no	no	no	no	3	3+4

B35	S2	F1	No subtype	No subtype	No subtype	no	no	no	no	no	no	no	no	no	3	3+4
B35	S3	F2	No subtype	No subtype	No subtype	no	no	no	no	no	no	no	no	no	3	4+3
B36	S1	F1	No subtype	No subtype	No subtype	no	no	no	no	no	no	no	no	no	3	4+3+5
B36	S2	F2	No subtype	No subtype	No subtype	no	no	no	no	no	no	no	no	no	3	3+4
B36	S3	F2	No subtype	No subtype	No subtype	no	no	no	no	no	no	no	no	no	3	3+4
B37	S1	F1	No subtype	No subtype	No subtype	no	no	no	no	no	no	no	no	no	3	4+3+5
B37	S2	F2	No subtype	No subtype	No subtype	no	no	no	no	no	no	no	no	no	3	3+4
B37	S3	F3	No subtype	No subtype	No subtype	no	no	no	no	no	no	no	no	no	3	3+4
B38	S1	F1	No subtype	No subtype	No subtype	no	no	no	no	no	no	no	no	no	2	3+4+5
B38	S2	F2	No subtype	No subtype	No subtype	no	no	no	no	no	no	no	no	no	2	3+4
B39	S1	F1	No subtype	No subtype	No subtype	no	no	no	no	no	no	no	no	no	1	3+4
B39	S2	F2	No subtype	No subtype	No subtype	no	no	no	no	no	no	no	no	no	1	3+4

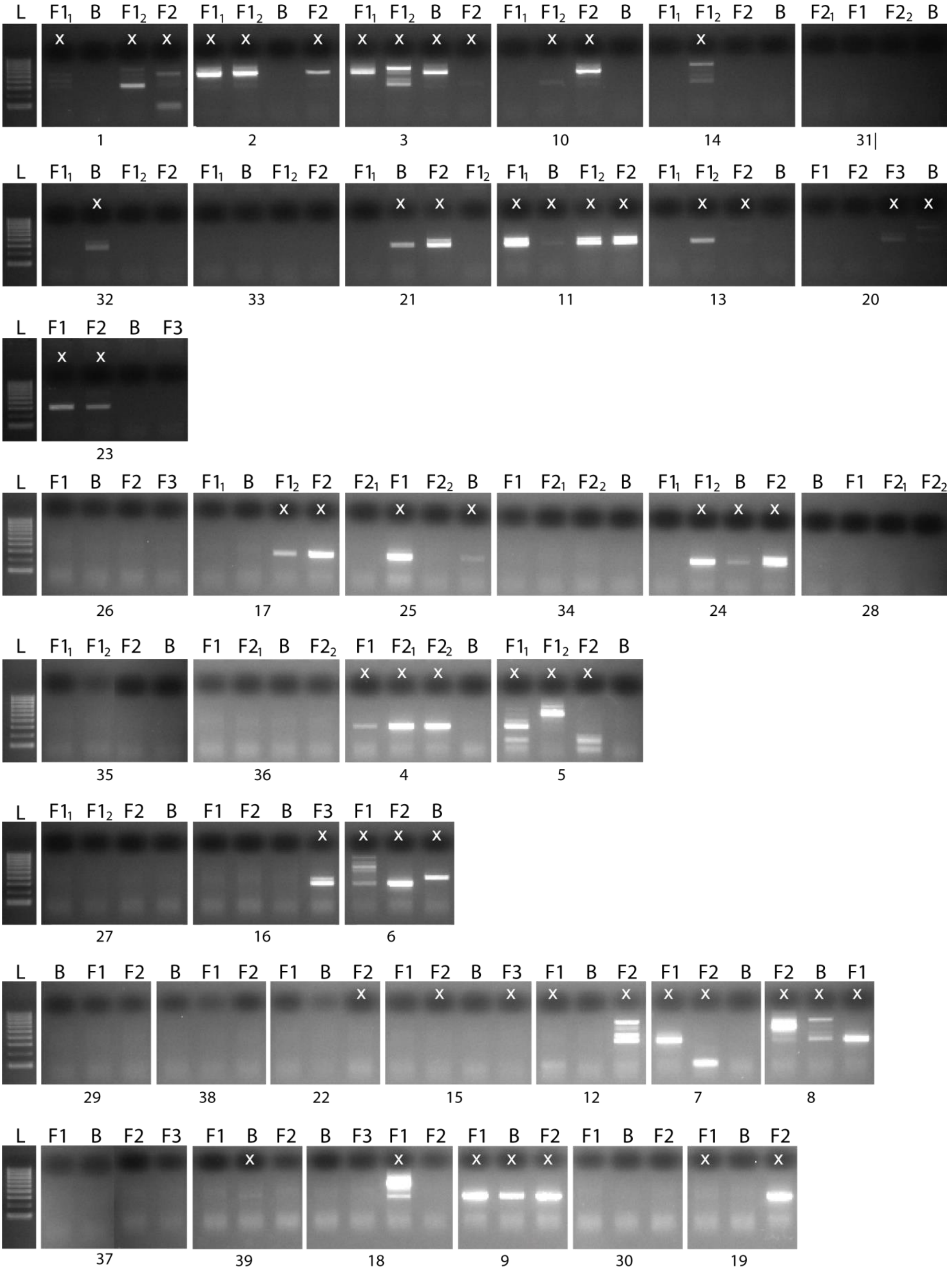

Supplementary Fig. S1. Visualization of fusion transcript *TMPRSS2-ERG.* UV-visualization of RT-PCR products from all samples (both tumor and benign) from all 39 patients, plus ladder. Primers were designed to pick up most known transcripts of *TMPRSS2-ERG* (Supplementary Table S1). L: 100 bp ladder, Neg: negative control, F1: focus 1, F2: focus 2, F3: focus 3, B: benign sample, x: detected fusion.

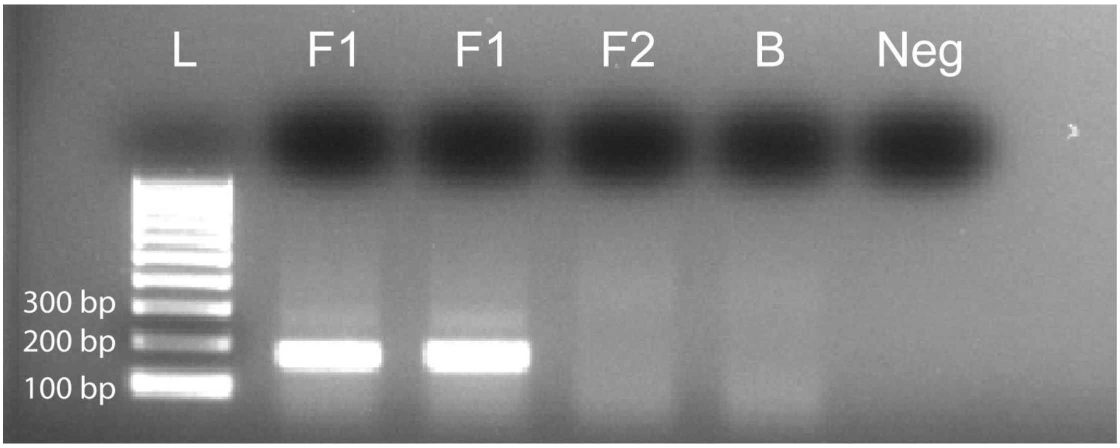

Supplementary Fig. S2. Validation of fusion transcript *ENSG00000263427-ERG*. UV-visualization of RT-PCR products from four samples plus ladder and one negative control. F1-1 and F1-2 both come from the same focus in one patient. L: 100 bp ladder, Neg: negative control, F1: focus 1, F2: focus 2, B: benign sample.

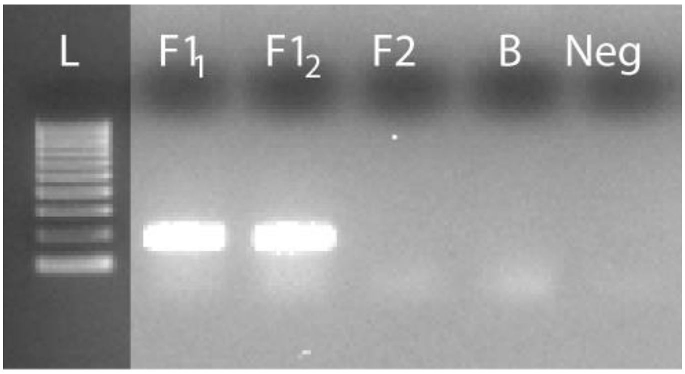

Supplementary Fig. S3. Validation of fusion transcript *TMPRSS2-ETV1*. UV-visualization of RT-PCR products from four samples plus ladder and one negative control. $F1_1$ and $F1_2$ both come from the same focus in one patient. L: 100 bp ladder, Neg: negative control, F1: focus 1, F2: focus 2, B: benign sample

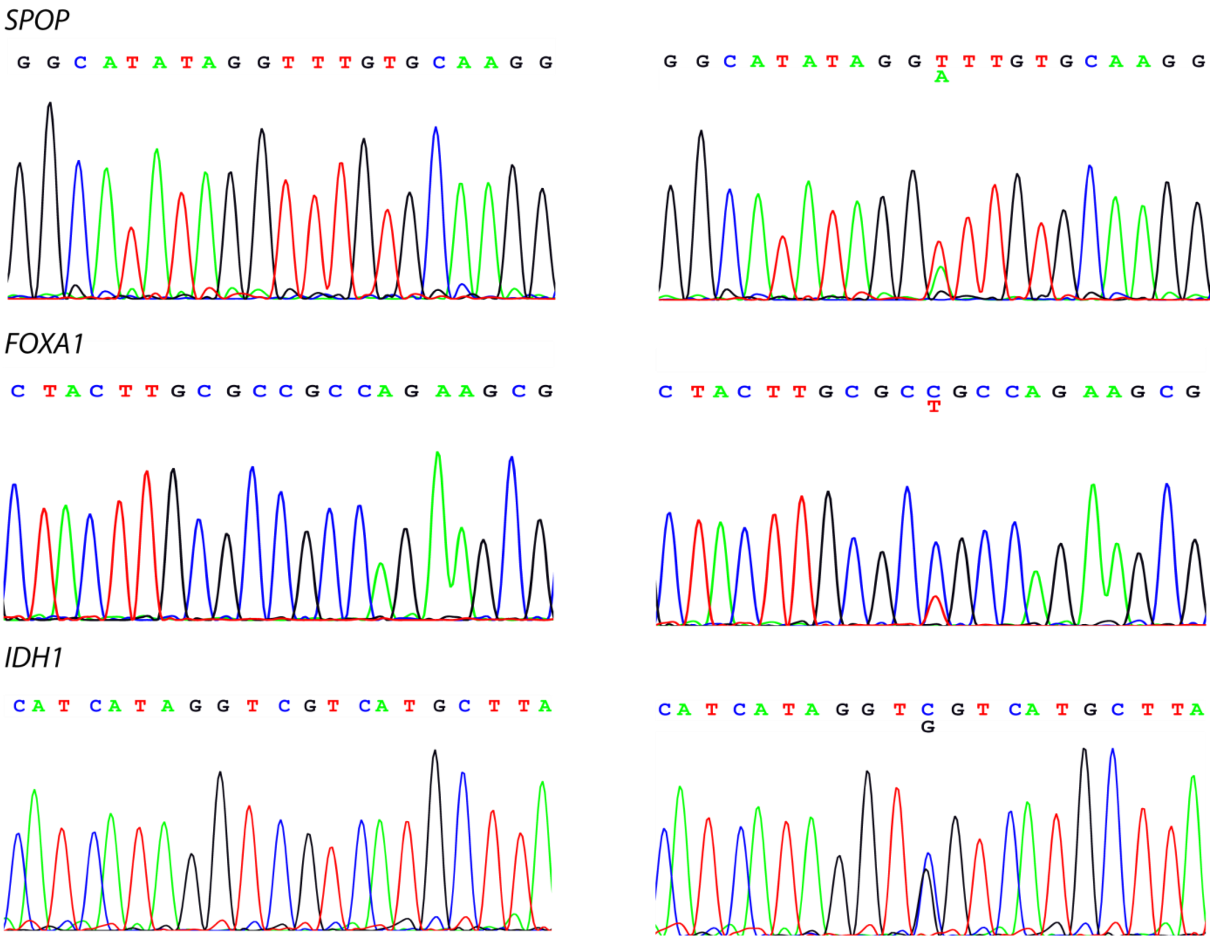

Supplementary Fig. S4. Validation of mutations included in the TCGA classification. Electropherograms after Sanger Sequencing, ten bases upstream and downstream of the mutation in genes *SPOP*, *FOXA1* and *IDH1*. Wild type sequence on the left side, mutated sequence on the right side.
